# Interplay Between Type 2 Diabetes Susceptibility and Prostate Cancer Progression: Functional Insights into *C2CD4A*

**DOI:** 10.3390/diagnostics15212767

**Published:** 2025-10-31

**Authors:** Yei-Tsung Chen, Chi-Fen Chang, Lih-Chyang Chen, Chao-Yuan Huang, Chia-Cheng Yu, Victor Chia-Hsiang Lin, Te-Ling Lu, Shu-Pin Huang, Bo-Ying Bao

**Affiliations:** 1 Department of Life Sciences and Institute of Genome Sciences, National Yang Ming Chiao Tung University, Taipei 112, Taiwan; yeitsungchen@nycu.edu.tw; 2 Department of Anatomy, School of Medicine, China Medical University, Taichung 406, Taiwan; cfchang@mail.cmu.edu.tw; 3 Department of Medicine, Mackay Medical College, New Taipei City 252, Taiwan; lihchyang@mmc.edu.tw; 4 Department of Urology, National Taiwan University Hospital, College of Medicine, National Taiwan University, Taipei 100, Taiwan; cyh0909@ntu.edu.tw; 5 Division of Urology, Department of Surgery, Kaohsiung Veterans General Hospital, Kaohsiung 813, Taiwan; ccyu@vghks.gov.tw; 6 Department of Urology, School of Medicine, National Yang-Ming University, Taipei 112, Taiwan; 7 Department of Pharmacy, College of Pharmacy and Health Care, Tajen University, Pingtung 907, Taiwan; 8 Department of Urology, E-Da Hospital, Kaohsiung 824, Taiwan; ed102161@edah.org.tw; 9 School of Medicine for International Students, I-Shou University, Kaohsiung 840, Taiwan; 10 Department of Pharmacy, China Medical University, Taichung 404, Taiwan; lutl@mail.cmu.edu.tw; 11 Graduate Institute of Clinical Medicine, College of Medicine, Kaohsiung Medical University, Kaohsiung 807, Taiwan; 12 Department of Urology, Kaohsiung Medical University Hospital, Kaohsiung 807, Taiwan; 13 Institute of Medical Science and Technology, College of Medicine, National Sun Yat-Sen University, Kaohsiung 804, Taiwan

**Keywords:** prostate cancer, recurrence, type 2 diabetes, C2CD4A, prognostic biomarker

## Abstract

**Background/Objective:** Biochemical recurrence (BCR) after radical prostatectomy (RP) for prostate cancer indicates disease progression. Although type 2 diabetes mellitus (T2D) shows a paradoxical association with prostate cancer risk, the prognostic role of T2D-related genetic variants remains unclear. **Methods:** We analyzed 113 common T2D susceptibility-related single-nucleotide polymorphisms (SNPs) in 644 Taiwanese men with localized prostate cancer (D’Amico risk classification: 12% low, 34% intermediate, and 54% high) treated with RP. Associations between SNPs and BCR were assessed using Cox regression, adjusting for key clinicopathological factors. Functional annotation was performed using HaploReg and FIVEx, while The Cancer Genome Atlas transcriptomic data were analyzed for C2 calcium-dependent domain-containing 4A (*C2CD4A*) expression. Gene set enrichment analysis (GSEA) and gene set variation analysis (GSVA) were applied to explore related biological pathways. **Results:** *C2CD4A* SNP *rs4502156* was independently associated with a reduced risk of BCR (hazard ratio = 0.80, *p* = 0.035). The protective *C* allele correlated with higher *C2CD4A* expression. Low *C2CD4A* expression is associated with advanced pathological stages, higher Gleason scores, and disease progression. GSEA revealed negative enrichment of mitotic and chromatid segregation pathways in high-*C2CD4A*-expressing tumors, with E2F targets being the most suppressed. GSVA confirmed an inverse correlation between *C2CD4A* expression and E2F pathway activity, with *CDKN2C* as a co-expressed functional gene. **Conclusions:** The T2D-related variant *rs4502156* in *C2CD4A* independently predicts a lower risk of BCR, potentially via suppression of the E2F pathway, and may serve as a germline biomarker for postoperative risk stratification.

## 1. Introduction

Prostate cancer is one of the most commonly diagnosed malignancies among men worldwide [1]. Although radical prostatectomy (RP) and radiotherapy achieve excellent oncological control of localized disease, biochemical recurrence (BCR) remains a major clinical challenge [2]. Current risk stratification models based on prostate-specific antigen (PSA) level, Gleason grade, pathological stage, and validated genomic classifiers such as Decipher or Oncotype DX [3,4] have improved prognostic accuracy but still misclassify a subset of patients, resulting in both overtreatment of indolent tumors and undertreatment of aggressive disease. Therefore, the identification of additional germline or molecular biomarkers that can refine postoperative risk assessment is of considerable clinical importance [5].

Epidemiological evidence suggests that type 2 diabetes mellitus (T2D) has a complex and paradoxical relationship with prostate cancer. Multiple large-scale studies and meta-analyses have indicated that T2D is associated with a modest reduction in the incidence of prostate cancer, particularly early-stage disease [6,7]. However, patients with T2D who develop prostate cancer often present with more aggressive tumors and experience poorer outcomes, including a higher risk of cancer progression and related death [8,9]. These observations may be explained by the metabolic and hormonal alterations characteristic of T2D, such as insulin resistance, hyperinsulinemia, chronic inflammation, and reduced testosterone levels, which can influence tumor development and progression in different ways [10]. Recent studies have elucidated several key molecular pathways linking T2D to prostate cancer progression. Hyperinsulinemia in T2D increases circulating insulin-like growth factor (IGF)-1 levels, activating downstream signaling cascades that enhance prostate cancer cell proliferation, survival, and metastasis via augmented glucose uptake and suppression of apoptosis [11,12]. Chronic inflammation, driven by T2D-associated hyperglycemia and insulin resistance, induces pro-inflammatory cytokines such as tumor necrosis factor (TNF)-α and interleukin (IL)-6, activates nuclear factor (NF)-κB, and generates reactive oxygen species. These processes collectively promote a tumor microenvironment conducive to invasiveness, epithelial–mesenchymal transition, and therapy resistance [13,14]. Moreover, phosphatidylinositol 3-kinase/protein kinase B/mammalian target of rapamycin signaling, often hyperactivated by insulin and IGF-1 in the diabetic state, contributes to the metabolic reprogramming of prostate cancer cells, inhibition of autophagy, and progression to castration-resistant disease by coordinating nutrient sensing with unchecked growth and anti-apoptotic responses [15,16]. Genome-wide association studies (GWASs) have identified hundreds of common variants that contribute to T2D susceptibility, highlighting the highly polygenic nature of this metabolic condition [17]. The genotypic risk scores of multiple single-nucleotide polymorphisms (SNPs), which aggregate the effects of these loci, have proven useful in predicting T2D risk but have shown minimal direct association with prostate cancer incidence [18,19]. Currently, there is limited evidence regarding the impact of T2D-related germline variants on cancer progression. Understanding whether such variants modulate disease recurrence could provide novel insights into the metabolic–genetic interplay between these two conditions and reveal new biomarkers for precise risk stratification.

This study investigated the association between established T2D susceptibility loci and BCR in men with localized prostate cancer treated with RP. By integrating genetic and clinical data, we aimed to identify metabolism-related genetic markers that may enhance postoperative prognostic assessments and guide individualized management strategies.

## 2. Patients and Methods

### 2.1. Patient and Response Assessment

In total, 644 men with histologically confirmed prostate cancer who underwent RP at National Taiwan University Hospital, Kaohsiung Medical University Hospital, and Kaohsiung Veterans General Hospital were enrolled in this study [20,21]. Institutional Review Board approval was obtained from Kaohsiung Medical University Hospital (KMU-HIRB-2013132), and all participants provided written informed consent. This study complied with the Declaration of Helsinki and the Good Clinical Practice guidelines. All participants were unrelated Han Taiwanese men diagnosed by systematic prostate biopsy, prompted by elevated PSA levels (>4 ng/mL) or abnormal findings on digital rectal examination during evaluation for lower urinary tract symptoms. Disease stage was defined according to the American Joint Committee on Cancer TNM classification based on pathological examination, pelvic computed tomography or magnetic resonance imaging, and radionuclide bone scans—only patients with clinically localized or locally advanced prostate cancer who underwent RP as primary treatment were included. Exclusion criteria included the receipt of neoadjuvant or adjuvant androgen deprivation therapy or radiotherapy before PSA recurrence, incomplete clinicopathological data, or insufficient postoperative follow-up (less than two months). After applying these criteria, 644 patients were included in the final analyses. Clinical and pathological data, including age, PSA level at diagnosis, pathological stage, Gleason score, surgical margin status, and lymph node metastasis, were retrieved from medical records and pathology reports. BCR was defined as two consecutive PSA measurements of 0.2 ng/mL or more after RP [22,23]. BCR-free survival was calculated from the date of surgery until BCR or the last follow-up. During the median follow-up period of 51 months, 229 (35.6%) patients achieved BCR (Table 1). Elevated PSA levels at diagnosis, advanced pathological stage, higher Gleason scores, higher International Society of Urological Pathology grades, high D’Amico risk classification, and positive surgical margins were significantly associated with BCR (*p* < 0.05) [24].

### 2.2. SNP Selection and Genotyping

In total, 139 common T2D-associated SNPs previously identified in a large-scale GWAS meta-analysis (62,892 patients with T2D and 596,424 healthy controls) were initially selected [25]. Genomic DNA was extracted from peripheral blood samples using the QIAamp DNA Blood Kit (Qiagen, Taipei City 100, Taiwan) and genotyped at the National Center for Genome Medicine using the Affymetrix Axiom platform (Thermo Fisher Scientific, Taichung 401, Taiwan) [26]. Variants with a minor allele frequency (MAF) of less than 0.03 and call rate of less than 0.95 or deviating from Hardy–Weinberg equilibrium (*p* < 1 × 10^−4^) were excluded, leaving 113 SNPs for final analysis.

### 2.3. Bioinformatic Analyses

To assess the regulatory potential of *rs4502156*, an SNP in *C2 calcium-dependent domain-containing 4A* (*C2CD4A*), HaploReg v4.2 was used to evaluate its effects on chromatin states, transcription factor-binding motifs, and evolutionary conservation [27]. Expression quantitative trait locus (eQTL) associations between the *rs4502156* genotype and *C2CD4A* expression were examined using the FIVEx database and linear regression [28]. To determine the clinical relevance of *C2CD4A* and *cyclin-dependent kinase 4 inhibitor C* (*CDKN2C*), their expression profiles were analyzed using The Cancer Genome Atlas prostate adenocarcinoma (TCGA-PRAD) dataset (497 primary tumors and 52 normal tissues, with no overlap with our cohort) via the Genomic Data Commons Data Portal.

### 2.4. Differential Gene Expression and Gene Enrichment Analyses

To uncover the molecular mechanisms linked to *C2CD4A*, Gene Ontology (GO) and Hallmark pathway assessments were performed using gene set enrichment analysis (GSEA) [29]. Patients in the TCGA-PRAD cohort were first categorized into high- and low-expression groups based on the median *C2CD4A* expression. Differential gene expression between groups was computed using the R limma package (v3.64.1). The resulting log-fold change rankings were analyzed via GSEA for GO biological processes and Hallmark sets using clusterProfiler (v4.16.0) in R. The five leading pathways were visualized using enrichplot (v1.28.2).

### 2.5. Gene Set Variation Analysis

Gene set variation analysis (GSVA) converts gene expression profiles across samples into pathway enrichment metrics, enabling the detection of subtle variations in extensive gene collections [30]. The GSVA package (v2.2.0) in R was used to derive the E2F target scores for each TCGA-PRAD specimen.

### 2.6. Statistical Analyses

All statistical analyses were performed using R (v4.5.1) and Statistical Product and Service Solutions (v19.0.0; IBM), with a two-tailed *p*-value of < 0.05 indicating statistical significance. Survival differences were assessed using Kaplan–Meier curves and log-rank tests. Cox proportional hazards models (univariate and multivariate) were used to evaluate the links between clinical features and outcomes, yielding hazard ratios (HRs) and 95% confidence intervals (CIs). Relationships between *C2CD4A* and *CDKN2C* expression levels, E2F GSVA scores, and cancer attributes were determined using Spearman and Pearson correlation tests.

## 3. Results

**Figure 1:**
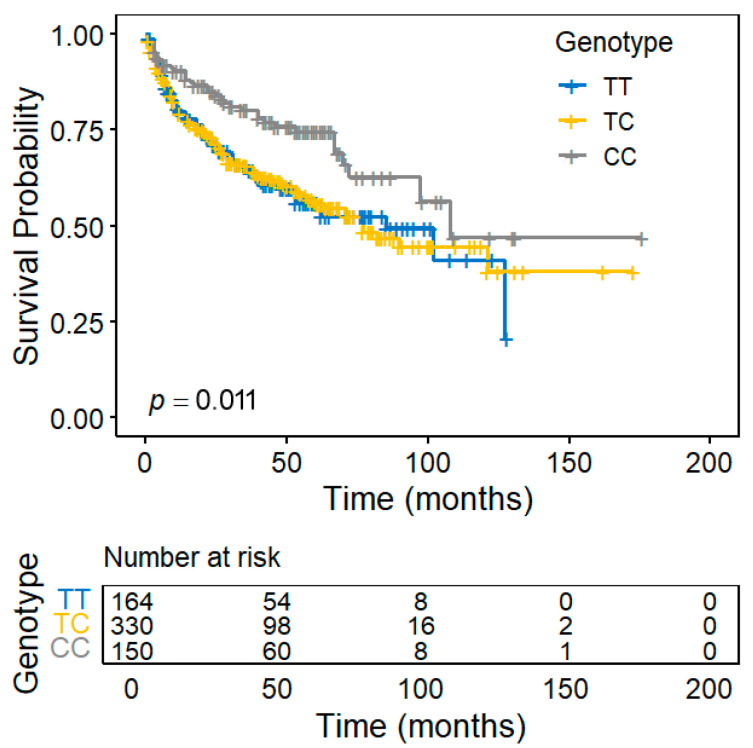
Kaplan–Meier curves of biochemical recurrence-free survival following radical prostatectomy, stratified by *rs4502156* genotypes in *C2CD4A*.

To investigate the relationship between T2D-related genetic variants and prostate cancer progression, we evaluated the association of 113 established T2D susceptibility-related SNPs with BCR following RP in patients with prostate cancer. Two SNPs, *rs11098676* in *nudix hydrolase 6* (*NUDT6*) and *rs4502156* in *C2CD4A*, were associated with BCR (*p* < 0.05; Table 2). In the multivariate Cox regression analysis adjusted for age, PSA level at diagnosis, pathological stage, Gleason score, surgical margin status, and lymph node metastasis, *rs4502156* remained independently significant (Figure 1). Moreover, each additional minor *C* allele of *rs4502156* conferred a 20% reduction in BCR risk (HR = 0.80; 95% CI = 0.65–0.98; *p* = 0.035).

**Figure 2:**
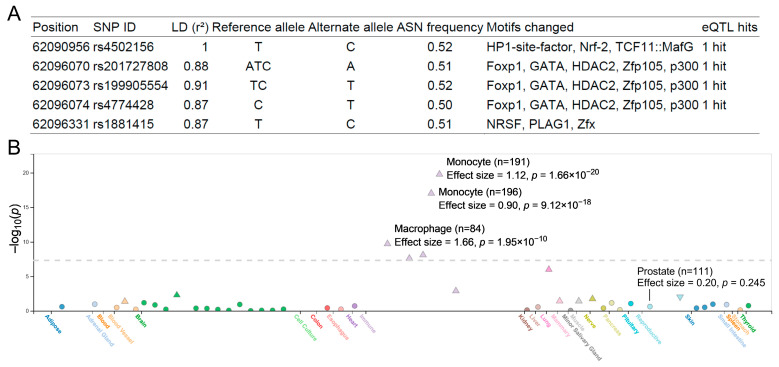
Functional characterization of the *C2CD4A rs4502156*. (**A**) Regulatory annotation from HaploReg showing that *rs4502156* and its linkage disequilibrium proxies are expression quantitative trait loci (eQTLs) that could potentially disrupt multiple transcription factor-binding motifs. (**B**) Tissue-wide eQTL identified from the FIVEx database. The dashed horizontal line indicates the genome-wide significance threshold (*p* = 5 × 10^−8^). Data points indicate the direction and significance of the association: positive (triangles), negative (inverted triangles), and nonsignificant (circles).

HaploReg analysis of *rs4502156* annotated the variant and its linkage disequilibrium proxies as eQTLs that could potentially disrupt several transcription factor-binding motifs (Figure 2A). The FIVEx database analysis corroborated this finding, indicating that the protective *C* allele was strongly associated with increased *C2CD4A* expression in multiple immune cell types, particularly monocytes and macrophages (*p* < 5 × 10^−8^; Figure 2B). However, this association was not statistically significant in prostate tissue (*p* = 0.245).

**Figure 3:**
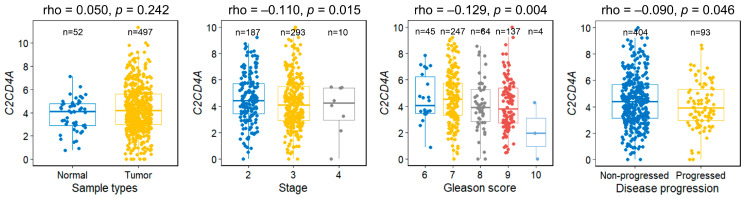
Association between *C2CD4A* mRNA expression and clinicopathological features of prostate cancer. Expression values (log2(x+1)-transformed RNA-Seq by expectation-maximization counts) were analyzed against the tumor stage, Gleason score, and disease progression status using data from The Cancer Genome Atlas prostate adenocarcinoma cohort. Lower *C2CD4A* expression was significantly associated with more aggressive disease.

To determine the clinical significance of *C2CD4A* expression in prostate cancer, we analyzed transcriptomic data from 497 tumors and 52 normal tissues in TCGA-PRAD. *C2CD4A* mRNA levels did not differ significantly between tumorous and normal tissues (Figure 3). Nevertheless, lower *C2CD4A* expression was significantly associated with a more advanced pathological stage, a higher Gleason score, and disease progression (*p* ≤ 0.046). *C2CD4A* expression showed modest discriminative ability in predicting disease progression, with an area under the receiver operating characteristic curve (AUC) of 0.57. This association was attenuated after adjustment for age, PSA level, pathological stage, and Gleason score (HR = 0.95; 95% CI = 0.85–1.06; *p* = 0.357).

**Figure 4:**
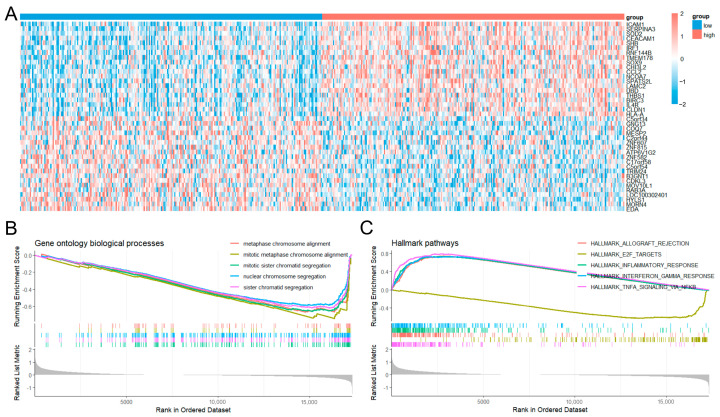
Gene Ontology annotation and pathway enrichment of genes associated with *C2CD4A* expression in prostate cancer. (**A**) Heatmap of the top 20 upregulated and downregulated differentially expressed genes between the high- and low-*C2CD4A*-expression groups in The Cancer Genome Atlas prostate adenocarcinoma cohort. (**B**) GSEA of Gene Ontology biological processes, highlighting the top five pathways enriched in the high-expression group. (**C**) GSEA of Hallmark pathways showing the top five enriched pathways, with E2F targets being the most significantly enriched gene set.

We further explored the biological role of *C2CD4A* by stratifying patients from TCGA-PRAD into high- and low-expression groups based on median expression levels (Figure 4A). GSEA of the ranked differential expression profiles revealed significant negative enrichment of genes involved in mitotic sister chromatid segregation, sister chromatid segregation, and nuclear chromosome segregation in the high expression group (Figure 4B). Consistent with this, Hallmark pathway analysis identified E2F targets as the most significantly downregulated pathway in the high-*C2CD4A*-expresion group (normalized enrichment score = −2.58, false discovery rate = 3.33 × 10^–10^; Figure 4C).

**Figure 5:**
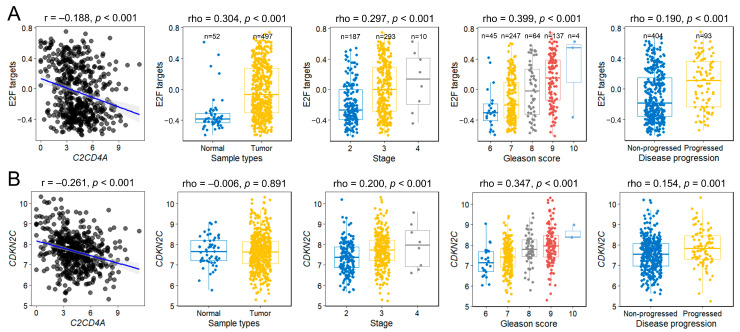
Clinical relevance of E2F pathway activity and *CDKN2C* expression in prostate cancer. (**A**) GSVA scores for E2F targets and (**B**) *CDKN2C* expression levels according to tumor stage, Gleason score, and disease progression status in The Cancer Genome Atlas prostate adenocarcinoma cohort. Both the E2F GSVA scores and *CDKN2C* expression levels were inversely correlated with the *C2CD4A* expression levels and elevated in more aggressive diseases. The blue regression line in scatter plots represents the best-fit linear model, and the shaded area indicates the 95% confidence interval.

Given this association, we quantified E2F pathway activity in each sample using GSVA. *C2CD4A* expression levels were inversely correlated with E2F GSVA scores (Figure 5A). E2F pathway activity was elevated in tumor tissues compared with normal tissues and was much higher in tumors at the advanced stage, with a high Gleason score and progressive disease (*p* < 0.001). The E2F GSVA scores showed discriminative ability in predicting disease progression, with an AUC of 0.64, and remained significant after adjustment for age, PSA, pathological stage, and Gleason score (HR = 3.01; 95% CI = 1.53–5.90; *p* = 0.01). To further elucidate this relationship, we examined the clinical relevance of *CDKN2C* (the most significantly differentially expressed E2F target gene between the high- and low-*C2CD4A*-expression groups). *C2CD4A* expression was inversely correlated with *CDKN2C* levels (Figure 5B). Although *CDKN2C* levels did not differ significantly between normal and tumorous tissues, its expression was higher in more aggressive tumors (*p* ≤ 0.001). The *CDKN2C* expression level also showed discriminative ability in predicting disease progression, with an AUC of 0.61, but the association was no longer significant after multivariable adjustment (HR = 1.20; 95% CI = 0.95–1.51; *p* = 0.134).

## 4. Discussion

In this multicenter cohort of Taiwanese men with localized prostate cancer, we identified a significant association between *rs4502156* (an SNP in the T2D-related *C2CD4A* gene) and a reduced risk of BCR following RP, independent of established clinicopathological factors. The protective *C* allele, which is linked to increased *C2CD4A* expression, is associated with more favorable tumor characteristics, including a lower pathological stage and Gleason score. In contrast, a T2D polygenic risk score constructed from 113 SNPs showed no significant association with BCR (HR = 0.94, 95% CI = 0.68–1.29, *p* = 0.681), suggesting that the aggregate genetic risk for T2D does not broadly influence prostate cancer recurrence. In contrast, specific loci such as rs4502156 may exert context-dependent effects. Bioinformatics analyses further suggested that elevated *C2CD4A* expression attenuates E2F target gene activity, which is critical for cell cycle regulation, potentially restraining tumor progression. These findings highlight a novel genetic–metabolic link in prostate cancer prognosis, expanding prior evidence of the paradoxical role of T2D in cancer incidence and aggressiveness. Although validation in an independent cohort would be ideal, these results should be interpreted as exploratory and promising, providing a preliminary framework for future mechanistic and clinical investigations.

The SNP *rs4502156*, which resides in the 3′ region of *C2CD4A*, was annotated as an eQTL and predicted to disrupt several transcription factor-binding motifs, including heterochromatin protein 1 (HP1), nuclear factor erythroid 2–related factor 2 (NRF2), transcription factor 11 (TCF11, also known as NRF1), and MAF bZIP transcription factor G (MafG). These disruptions may alter *C2CD4A* expression and its tumor-suppressive function in prostate cancer, which is potentially amplified by diabetes-related mechanisms. Specifically, disruption of the HP1-binding motif can impair heterochromatin maintenance, thereby modulating the epigenetic regulation of *C2CD4A*, as HP1 downregulation has been linked to genome instability in diabetes and cancer progression [31,32]. Similarly, alterations in the NRF2 motif may weaken antioxidant and detoxification responses, exacerbating oxidative stress—a hallmark of T2D—where NRF2 dysregulation promotes cancer survival via metabolic reprogramming [33,34]. Additionally, interference with the TCF11 and MafG motifs could disrupt heterodimeric regulation of proteasomal and stress-response genes, contributing to proteotoxic stress in T2D, as TCF11 plays a tumor-suppressive role in proteostasis and MafG, via its lncRNA axis, influences glucose metabolism [35,36]. eQTL analysis showed that the protective *C* allele was associated with increased *C2CD4A* expression across multiple immune cell subsets. In contrast, only a non-significant trend was observed in prostate tissue, likely due to the limited sample size. *C2CD4A* encodes a nuclear C2 domain-containing protein that is best known for its role in metabolic regulation, particularly within pancreatic β-cells where it functions as a transcriptional cofactor that enhances glycolytic gene expression while suppressing “disallowed” genes, with *C2cd4a*-knockout mice showing impaired glucose tolerance [37]. In cancer, the role of *C2CD4A* appears to be context-dependent. Elevated *C2CD4A* transcript levels have been documented in colon cancer, where higher gene expression correlates with an advanced disease stage [38,39]. Moreover, functional studies have shown that *C2CD4A* promotes tumor growth by binding to p53 and enhancing its ubiquitination and degradation, thereby suppressing apoptosis [40]. Conversely, in bladder cancer, *C2CD4A* upregulation has been linked to treatment with the anticancer compound isorhapontigenin, which induces cell cycle arrest and activates interferon signaling [41]. Although germline variants of *C2CD4A* have been well established to be associated with T2D susceptibility, their contribution to cancer risk remains poorly defined; only one case–control study reported a protective association between *C2CD4A/B* region variants and lung cancer risk [42].

GSEA of *C2CD4A*-associated expression networks identified a negative correlation between *C2CD4A* expression and that of E2F target genes, including *CDKN2C*. E2F transcription factors modulate the expression of genes critical for proliferation, DNA replication, and apoptosis, and their dysregulation is a hallmark of oncogenesis across tumor types [43]. CDKN2C (p18^INK4C^), a cyclin-dependent kinase (CDK) inhibitor belonging to the INK4 family, suppresses CDK4/6 activity and induces G1 arrest [44]. *CDKN2C* is frequently functionally inactivated in multiple malignancies; for instance, pan-cancer analyses have described decreased expression or deletion in diverse tumors [45]. Paradoxically, *CDKN2C* is overexpressed in certain cancer types, such as small-cell lung cancer, where elevated levels are associated with poor survival [45]. In prostate cancer, E2F-related gene signatures encompassing *CDKN2C* can be used to stratify recurrence risk, with higher signature expression predicting worse recurrence-free survival, which is consistent with net pathway activation despite the nominal inhibitory role of *CDKN2C* [46].

Furthermore, inflammation- and TNF-α–related pathways were also enriched in our *C2CD4A* GSEA. These pathways are known to converge on the cell cycle machinery and modulate E2F transcriptional activity via CDKs. TNF-α activates NF-κB, which transcriptionally induces cyclin D and CDK6; these cyclin D/CDK4/6 complexes phosphorylate the retinoblastoma (RB) protein, releasing E2F and promoting G1–S phase transition. Elevated E2F activity in tumors frequently arises from CDK overexpression and RB inactivation [47]. E2F factors also reciprocally modulate inflammatory signaling: in response to TNF-α, E2F1 physically interacts with the NF-κB subunit RelA, competing with NF-κB inhibitor-α for RelA binding, thereby influencing NF-κB nuclear translocation and fine-tuning the amplitude and timing of inflammatory gene expression. NF-κB targets include cyclin D and the CDK inhibitor p21, highlighting the intricate feedback between inflammation and cell-cycle control [48]. Beyond their canonical cell-cycle roles, CDK4/6 kinases exert immune-modulatory functions. In CD8⁺ T cells, CDK6 deficiency does not impair TNF-α or interferon-γ production but enhances IL-2 secretion, while CDK4/6 inhibition augments antigen presentation and cytokine production, demonstrating the dual role of these kinases in both proliferation and immune regulation [49]. Collectively, these observations underscore the complexity of the *C2CD4A*–E2F axis and warrant further investigation into its mechanistic role in prostate cancer progression and its potential as a prognostic or therapeutic target.

This study has several strengths. To our knowledge, this is the first study to evaluate T2D-associated genetic variants in relation to BCR in prostate cancer, with comprehensive clinicopathological adjustment. Second, a combination of genomic, transcriptomic, and pathway analyses allowed us to establish statistical associations and mechanistic insights, thereby providing a biologically coherent link between *C2CD4A* variants and disease progression. Third, the use of a well-defined homogenous Han Taiwanese cohort minimized the potential confounding effects of population stratification. Nevertheless, this study had several significant limitations that should be considered when interpreting the findings. First, the false discovery rate (FDR) for the identified SNP was relatively high (*p* = 0.011; Benjamini–Hochberg FDR = 0.760), suggesting a potential risk of type I error. Therefore, the association between rs4502156 and BCR should be viewed as exploratory until confirmed in independent cohorts. Second, the retrospective design may have introduced selection bias, and the modest sample size (*n* = 644) may have limited the power to detect small effect sizes or rare-variant associations. Power estimation suggested adequate detection for HRs ≤ 0.75 or ≥ 1.33 at α = 0.05, but the study was underpowered for weaker genetic effects. Third, the study population consisted exclusively of Taiwanese men of Han ethnicity, limiting the generalizability of the findings to other ethnic groups. Differences in allele frequencies and linkage disequilibrium patterns across populations may influence the strength or direction of genetic associations. Thus, external validation in multi-ethnic cohorts is required to confirm the clinical relevance of these findings. Fourth, the functional effects of rs4502156 were inferred primarily from bioinformatic annotations and expression correlation analyses rather than from direct laboratory validation. Although in silico analyses provided supportive evidence, the absence of tumor sequencing data in our cohort precluded direct genotype-expression integration, and TCGA data may not fully reflect post-radical prostatectomy disease biology. Finally, the follow-up duration, although sufficient for BCR assessment, did not allow the evaluation of long-term outcomes, such as cancer-specific survival. Consequently, the prognostic implications of rs4502156, beyond early recurrence, remain unclear. Collectively, these limitations underscore the need for larger, prospectively designed, multicenter studies that incorporate experimental validation and extended clinical follow-up to substantiate the current findings and clarify their translational potential.

## 5. Conclusions

In conclusion, we have elucidated a mechanistic link between genetic susceptibility to T2D and the progression of prostate cancer, mediated by *C2CD4A* modulation of the E2F pathways, offering a potential biomarker for refined risk stratification post-RP. Future prospective studies on diverse populations are warranted to validate the prognostic utility of *rs4502156* and to explore the therapeutic targeting of *C2CD4A*–E2F interactions. Although the present findings await validation in an independent cohort, they offer an exploratory yet compelling signal that supports further research into the metabolic–genomic interplay underlying prostate cancer progression. Importantly, the clinical implications of *rs4502156* and *C2CD4A* should be considered complementary to, rather than a replacement for, established genomic classifiers, offering an additional layer of prognostic information. Integrating such germline variants into existing nomograms could enhance personalized disease management and reduce overtreatment, while identifying high-risk patients for intensified surveillance or adjuvant therapies, ultimately improving outcomes in patients with prostate cancer.

## Figures and Tables

**Table 1:** Clinicopathologic characteristics of the study population.

Characteristics	No BCR	BCR	*p*
No. of patients, n (%)	415 (64.4)	229 (35.6)	
Median age at diagnosis, years (IQR)	66 (62–70)	66 (61–71)	0.299
Median PSA at diagnosis, ng/mL (IQR)	9.3 (6.2–15.0)	14.7 (8.4–25.9)	<0.001
Pathologic stage, n (%)			
T1/T2	275 (66.4)	89 (39.6)	<0.001
T3/T4/N1	139 (33.6)	136 (60.4)	
Pathologic Gleason score, n (%)			
2–7	369 (88.4)	164 (71.6)	<0.001
8–10	48 (11.6)	65 (28.4)	
ISUP grade group			
1	117 (28.2)	36 (15.7)	<0.001
2 and 3	250 (60.2)	128 (55.9)	
4	22 (5.3)	20 (8.7)	
5	26 (6.3)	45 (19.7)	
D’Amico risk classification, n (%)			
Low	65 (15.7)	12 (5.3)	<0.001
Intermediate	166 (40.1)	50 (21.9)	
High	183 (44.2)	166 (72.8)	
Surgical margin, n (%)			
Negative	320 (77.1)	140 (61.1)	<0.001
Positive	95 (22.9)	89 (38.9)	
Lymph node metastasis, n (%)			
Negative	323 (95.8)	182 (95.3)	0.763
Positive	14 (4.2)	9 (4.7)	

Abbreviations: BCR, biochemical recurrence; IQR, interquartile range; PSA, prostate-specific antigen; ISUP, International Society of Urological Pathology. The median follow-up duration was 51 months. Subtotals may not sum to the total number of patients due to missing data.

**Table 2:** Association of type 2 diabetes-related genetic variants with biochemical recurrence after radical prostatectomy.

SNP ID	Chromosome	Position	Mapped Gene	Allele	MAF	HWE	Risk Allele for T2D	Risk Allele for BCR	HR	*p*
*rs2296173*	1	39913351	*MACF1*	*A>G*	0.166	1.000	*G*	*A*	0.876	0.317
*rs12088739*	1	51506886	*MIR4421*	*A>G*	0.100	0.664	*A*	*A*	0.804	0.197
*rs1127655*	1	117530507	*PTGFRN*	*C>T*	0.438	0.080	*C*	*T*	1.161	0.093
*rs340874*	1	214159256	*PROX1-AS1*	*T>C*	0.387	0.282	*C*	*C*	1.018	0.859
*rs2820426*	1	219660535	*LOC102723886 (LYPLAL1)*	*A>G*	0.330	0.426	*G*	*A*	0.898	0.299
*rs348330*	1	229672955	*ABCB10*	*G>A*	0.435	0.130	*G*	*G*	0.883	0.212
*rs2867125*	2	622827	*TMEM18*	*C>T*	0.078	0.165	*C*	*T*	1.028	0.875
*rs780094*	2	27741237	*GCKR*	*T>C*	0.492	0.814	*C*	*T*	0.969	0.737
*rs243019*	2	60585806	*MIR4432HG*	*C>T*	0.345	0.603	*C*	*C*	0.853	0.109
*rs1009358*	2	65276452	*CEP68*	*T>C*	0.284	0.441	*T*	*T*	0.976	0.822
*rs10169613*	2	111934977	*BCL2L11*	*C>T*	0.455	0.692	*C*	*T*	1.038	0.685
*rs12617659*	2	121309759	*LOC105373585 (GLI2)*	*C>T*	0.184	0.192	*C*	*C*	0.918	0.480
*rs7572970*	2	161136656	*RBMS1*	*G>A*	0.185	0.605	*G*	*G*	0.995	0.968
*rs13389219*	2	165528876	*COBLL1*	*C>T*	0.098	0.501	*C*	*C*	0.830	0.286
*rs2972144*	2	227101411	*MIR5702*	*G>A*	0.064	1.000	*G*	*A*	1.129	0.518
*rs7561798*	2	228973660	*SPHKAP*	*G>A*	0.325	0.858	*G*	*A*	1.075	0.480
*rs1899951*	3	12394840	*PPARG*	*C>T*	0.053	0.701	*C*	*T*	1.081	0.710
*rs1496653*	3	23454790	*UBE2E2*	*A>G*	0.213	1.000	*A*	*G*	1.132	0.276
*rs11926707*	3	46925539	*PTH1R*	*C>T*	0.359	0.932	*C*	*C*	0.961	0.684
*rs2292662*	3	63897215	*ATXN7*	*C>T*	0.424	0.520	*C*	*T*	1.091	0.354
*rs6795735*	3	64705365	*ADAMTS9-AS2*	*T>C*	0.242	0.915	*C*	*T*	0.812	0.067
*rs4472028*	3	152053250	*MBNL1*	*C>T*	0.438	1.000	*T*	*T*	1.107	0.285
*rs11925227*	3	170766618	*TNIK*	*G>A*	0.169	0.576	*G*	*G*	0.901	0.423
*rs7651090*	3	185513392	*IGF2BP2*	*A>G*	0.249	0.599	*G*	*A*	1.000	0.999
*rs3887925*	3	186665645	*ST6GAL1*	*C>T*	0.475	0.157	*T*	*C*	0.952	0.591
*rs1801214*	4	6303022	*WFS1*	*T>C*	0.077	0.045	*T*	*C*	1.113	0.490
*rs17086692*	4	53134293	*SPATA18*	*G>T*	0.257	0.918	*G*	*T*	1.145	0.196
*rs993380*	4	83584496	*SCD5*	*G>A*	0.364	0.735	*A*	*A*	1.120	0.233
*rs7674212*	4	103988899	*SLC9B2*	*G>T*	0.382	0.868	*G*	*T*	1.130	0.209
*rs11098676*	4	123833154	*NUDT6*	*C>T*	0.039	1.000	*C*	*C*	0.558	0.047
*rs7685296*	4	153254121	*TMEM154*	*C>T*	0.442	1.000	*C*	*T*	1.035	0.713
*rs1061813*	5	14847331	*ANKH*	*A>G*	0.182	0.012	*G*	*A*	0.854	0.225
*rs4865796*	5	53272664	*ARL15*	*A>G*	0.117	0.124	*A*	*G*	1.024	0.867
*rs459193*	5	55806751	*C5orf67*	*G>A*	0.499	0.387	*G*	*A*	1.122	0.235
*rs2307111*	5	75003678	*POC5*	*C>T*	0.431	0.576	*T*	*T*	1.175	0.089
*rs6878122*	5	76427311	*ZBED3-AS1*	*A>G*	0.060	0.499	*G*	*A*	0.975	0.898
*rs10077431*	5	112927686	*YTHDC2*	*C>A*	0.101	0.829	*C*	*A*	1.296	0.064
*rs1050226*	6	7281654	*RREB1*	*A>G*	0.414	0.808	*A*	*G*	1.046	0.623
*rs7756992*	6	20679709	*CDKAL1*	*A>G*	0.482	0.432	*G*	*A*	0.992	0.934
*rs2857605*	6	31524851	*NFKBIL1*	*T>C*	0.156	0.072	*T*	*C*	1.250	0.066
*rs1063355*	6	32627714	*HLA-DQB1*	*G>T*	0.329	0.656	*G*	*G*	0.840	0.089
*rs9369425*	6	43810974	*LOC107986598 (VEGFA)*	*A>G*	0.144	0.265	*G*	*G*	1.191	0.177
*rs72892910*	6	50816887	*TFAP2B*	*G>T*	0.134	0.173	*T*	*T*	1.252	0.074
*rs853974*	6	127068983	*RPS4XP9*	*C>T*	0.468	1.000	*T*	*T*	1.014	0.883
*rs2246012*	6	131898208	*ARG1, MED23*	*T>C*	0.402	0.327	*C*	*T*	0.967	0.722
*rs622217*	6	160766770	*SLC22A3*	*T>C*	0.296	0.258	*T*	*T*	0.918	0.410
*rs17168486*	7	14898282	*DGKB*	*C>T*	0.488	0.529	*T*	*C*	0.940	0.526
*rs2191348*	7	15064255	*AGMO*	*T>G*	0.311	0.000	*T*	*T*	0.836	0.107
*rs2908282*	7	44248828	*YKT6*	*G>A*	0.194	1.000	*A*	*G*	0.933	0.569
*rs2299383*	7	103418846	*RELN*	*C>T*	0.404	0.807	*T*	*C*	0.942	0.524
*rs13239186*	7	117510621	*CTTNBP2*	*C>T*	0.188	0.438	*T*	*T*	1.060	0.617
*rs13234269*	7	130429186	*LOC105375508 (KLF14)*	*T>A*	0.327	0.789	*T*	*A*	1.033	0.750
*rs11774915*	8	9188762	*LOC157273(TNKS)*	*C>T*	0.316	0.364	*T*	*T*	1.040	0.684
*rs10100265*	8	10633159	*PINX1*	*A>C*	0.357	0.494	*A*	*A*	0.900	0.296
*rs17411031*	8	19852310	*LPL*	*C>G*	0.187	1.000	*C*	*C*	0.848	0.198
*rs10087241*	8	30863722	*PURG*	*A>G*	0.033	1.000	*G*	*G*	1.108	0.691
*rs12681990*	8	36859186	*KCNU1*	*T>C*	0.343	0.029	*C*	*T*	0.961	0.675
*rs516946*	8	41519248	*ANK1*	*C>T*	0.130	0.484	*C*	*T*	1.215	0.139
*rs7845219*	8	95937502	*TP53INP1*	*C>T*	0.281	0.923	*T*	*C*	0.960	0.699
*rs3802177*	8	118185025	*SLC30A8*	*G>A*	0.461	0.134	*G*	*A*	1.013	0.890
*rs2294120*	8	146003567	*ZNF34*	*G>A*	0.286	0.923	*A*	*A*	1.138	0.198
*rs10974438*	9	4291928	*GLIS3*	*A>C*	0.378	0.616	*C*	*A*	0.950	0.596
*rs1063192*	9	22003367	*CDKN2B-AS1/CDKN2B*	*A>G*	0.180	0.351	*A*	*G*	1.046	0.715
*rs10811661*	9	22134094	*CDKN2B-AS1*	*T>C*	0.408	0.626	*T*	*C*	1.076	0.432
*rs1758632*	9	34025640	*UBAP2*	*G>C*	0.156	0.100	*G*	*C*	1.038	0.783
*rs17791483*	9	81898980	*LOC101927450 (TLE1)*	*A>G*	0.052	1.000	*A*	*G*	1.180	0.424
*rs2796441*	9	84308948	*LOC101927502 (TLE1)*	*A>G*	0.384	0.803	*G*	*A*	0.927	0.433
*rs10114341*	9	96919182	*LOC107987099 (PTPDC1)*	*T>C*	0.090	0.338	*T*	*T*	0.767	0.148
*rs687621*	9	136137065	*ABO*	*A>G*	0.340	0.541	*G*	*G*	1.028	0.787
*rs11257655*	10	12307894	*CDC123*	*T>C*	0.449	0.000	*T*	*T*	0.994	0.949
*rs2616132*	10	71469514	*FAM241B*	*G>A*	0.484	0.814	*A*	*A*	1.075	0.436
*rs753270*	10	80964975	*ZMIZ1*	*T>C*	0.455	0.812	*C*	*C*	1.082	0.389
*rs11591741*	10	101976501	*CHUK*	*G>C*	0.063	0.100	*G*	*G*	0.974	0.897
*rs2421016*	10	124167512	*PLEKHA1*	*C>T*	0.433	0.066	*C*	*C*	0.934	0.457
*rs2237892*	11	2839751	*KCNQ1*	*C>T*	0.340	0.485	*C*	*T*	1.003	0.974
*rs5215*	11	17408630	*KCNJ11*	*T>C*	0.374	0.276	*C*	*C*	1.084	0.414
*rs7929543*	11	49351026	*TYRL*	*A>C*	0.148	0.061	*C*	*A*	0.843	0.223
*rs1552224*	11	72433098	*ARAP1*	*A>C*	0.064	1.000	*A*	*C*	1.325	0.095
*rs10830963*	11	92708710	*MTNR1B*	*C>G*	0.431	0.690	*G*	*C*	0.919	0.366
*rs7931302*	11	128236058	*ETS1*	*A>C*	0.154	0.228	*C*	*A*	0.835	0.201
*rs67232546*	11	128398938	*ETS1*	*C>T*	0.172	0.679	*T*	*C*	0.811	0.114
*rs12299509*	12	4406281	*CCND2*	*G>A*	0.472	0.070	*G*	*A*	1.150	0.116
*rs11048456*	12	26463082	*ITPR2*	*C>T*	0.307	0.581	*C*	*C*	0.955	0.647
*rs10842994*	12	27965150	*LOC105369709 (KLHL42)*	*C>T*	0.205	0.811	*C*	*T*	1.111	0.367
*rs2261181*	12	66212318	*RPSAP52*	*C>T*	0.115	0.334	*T*	*T*	1.277	0.064
*rs1480474*	12	66326943	*HMGA2*	*G>A*	0.085	0.610	*G*	*G*	0.927	0.671
*rs7138300*	12	71439589	*TSPAN8*	*C>T*	0.370	0.556	*C*	*T*	1.055	0.592
*rs11107116*	12	93978504	*SOCS2*	*G>T*	0.330	0.248	*T*	*G*	0.995	0.956
*rs940904*	12	123491572	*PITPNM2*	*A>G*	0.138	0.136	*A*	*A*	0.953	0.722
*rs825476*	12	124568456	*ZNF664-FAM101A*	*T>C*	0.370	0.736	*T*	*T*	0.902	0.288
*rs576674*	13	33554302	*KL*	*A>G*	0.212	0.906	*G*	*A*	0.953	0.681
*rs963740*	13	51096095	*DLEU1*	*T>A*	0.344	0.543	*A*	*A*	1.096	0.339
*rs1359790*	13	80717156	*LOC105370275 (SPRY2)*	*G>A*	0.289	0.848	*G*	*A*	1.052	0.621
*rs4502156*	15	62383155	*C2CD4A*	*T>C*	0.488	0.637	*T*	*T*	0.788	0.011
*rs982077*	15	63823301	*USP3*	*A>G*	0.056	0.713	*A*	*G*	1.377	0.087
*rs7177055*	15	77832762	*LOC101929457 (HMG20A)*	*G>A*	0.333	0.002	*A*	*G*	0.913	0.343
*rs9940149*	16	300641	*FAM234A*	*G>A*	0.429	0.689	*G*	*G*	0.951	0.598
*rs7185735*	16	53822651	*FTO*	*A>G*	0.113	0.116	*G*	*G*	1.113	0.471
*rs244415*	16	69666683	*NFAT5*	*G>A*	0.105	1.000	*G*	*G*	0.925	0.603
*rs2925979*	16	81534790	*CMIP*	*C>T*	0.426	0.378	*T*	*C*	0.953	0.606
*rs8068804*	17	3985864	*ZZEF1*	*G>A*	0.160	0.306	*A*	*G*	0.895	0.386
*rs12945601*	17	17653411	*RAI1*	*T>C*	0.095	0.822	*T*	*C*	1.114	0.487
*rs11651755*	17	36099840	*HNF1B*	*T>C*	0.173	0.335	*C*	*T*	0.924	0.524
*rs9911983*	17	45885756	*OSBPL7*	*C>T*	0.157	0.767	*T*	*T*	1.056	0.665
*rs9894220*	17	46989154	*UBE2Z*	*A>G*	0.251	0.834	*A*	*A*	0.850	0.137
*rs7240767*	18	7070642	*LAMA1*	*C>T*	0.306	0.927	*C*	*C*	0.995	0.958
*rs12970134*	18	57884750	*MC4R*	*G>A*	0.156	0.765	*A*	*A*	1.253	0.068
*rs10401969*	19	19407718	*SUGP1*	*T>C*	0.105	0.529	*C*	*C*	1.221	0.172
*rs8108269*	19	46158513	*GIPR*	*T>G*	0.438	0.750	*G*	*T*	0.945	0.550
*rs6515236*	20	22435749	*LOC105372562 (FOXA2)*	*C>A*	0.333	0.185	*A*	*C*	0.883	0.232
*rs6059662*	20	32675727	*EIF2S2*	*G>A*	0.156	0.882	*G*	*G*	0.990	0.936
*rs4810426*	20	43001721	*HNF4A*	*C>T*	0.413	0.686	*T*	*T*	1.128	0.199
*rs4823182*	22	44377442	*SAMM50*	*G>A*	0.492	0.388	*G*	*G*	0.974	0.772

Abbreviations: SNP, single nucleotide polymorphism; MAF, minor allele frequency; HWE, Hardy–Weinberg equilibrium; T2D, type 2 diabetes mellitus; BCR, biochemical recurrence; HR, hazard ratio.

## Data Availability

Data will be available on reasonable request.
